# Smart Handovers, Safer Care in the NHS: Elevating MDT Performance in Notts Crisis and Home Treatment Teams

**DOI:** 10.1192/bjo.2026.11455

**Published:** 2026-06-30

**Authors:** Ann Panjikkaran, Shweta Mittal, Aman Sardana

**Affiliations:** 1 Sheffield Health Partnership University NHS Foundation Trust, Sheffield, United Kingdom; 2 Nottinghamshire Healthcare NHS Foundation Trust, Worksop, United Kingdom; 3 Sussex Partnership NHS Foundation Trust, Worthing, United Kingdom

## Abstract

**Aims:**

Clinical handovers are vital in the delivery of safe and effective patient care. It is central in relaying important patient information and professional responsibility between healthcare providers. The community-based Crisis Resolution and Home Treatment (CRHT)teams in Worksop conduct their handovers daily with duties distributed among different consultants on a rotating basis. Handovers are utilised for sharing pertinent updates regarding the patient’s journey through the crisis team. This allows for collaborative decision making with the wider team.

The aim of this project is to evaluate the effectiveness of handovers within the Mid and North CRHT by identifying structural and communication gaps in the multidisciplinary team (MDT) and suggesting targeted improvements to promote safe and high-quality patient care.

**Methods:**

To understand staff perspectives of how effective current handover processes in CRHT are, a pre-intervention Microsoft forms questionnaire was sent to all clinical members of the team. The questionnaire was anonymous and included Likert-scale questions on patient safety and MDT structure as well as free text for suggesting improvements. Following the collation of results, a meeting with the clinical lead and non-medical prescriber was conducted to discuss previously trialled methods to make handover processes more streamlined.

**Results:**

17 completed pre-intervention forms were received. 23% of staff ‘strongly agree’ that the current handover process ensures patient safety. 18% of staff ‘strongly agree’ that the current handover follows a structured process whereas 6% of staff ‘disagree’. Thematic analysis suggests staff want to enhance current handover by improving its structure and ensuring that individual patients are handed over by designated staff members who are familiar with the patient. The feedback from the questionnaires as well as the discussions from the meeting were used to inform new changes to the MDT.

**Conclusion:**

Staff feedback has provided valuable insight into the limitations of the current CRHT handover process. An amended standardised template and staff reminders to present familiar patients have been introduced. New changes include visible onward referrals and intended date of discharge in the documentation which hopes to track patient journey more accurately. Night shift staff who allocate patients for handover to designated morning staff have been advised to assign staff based on familiarity with patient’s presentation. Post intervention questionnaires will be sent to staff once sufficient time for the implemented changes to take full effect has lapsed.

